# Supplementary material for: How and Why Diets Change Post-Migration: A Qualitative Exploration of Dietary Acculturation among Recent Chinese Immigrants in Australia
Source: Nutrients. 2022 Aug 30;14(17):3573. doi: 10.3390/nu14173573 (PMC9460769; doi:10.3390/nu14173573)
Supplement: Supplementary file 1 [file nutrients-14-03573-s001.zip › nutrients-1871791-supplementary.pdf]

初期問卷Baseline Questionnaire

個人資料DEMOGRAPHIC

**1. Age (years) 年龄（多少年)**	

**2. Gender Identity 性别**
☐ Male 男
☐ Female **女**
☐ Other (please specify) 其他:

**3. Marital Status 婚姻状况**
☐ Single单身
☐ Married/Partner已婚
☐ Widowed丧偶
☐ Divorced离异
☐ Separated分居

**4. Number of household members (including yourself):** 包括你在内，你家里有多少人	

**5. Do you have dependent children? 你有被**抚养的孩子吗？
☐ Yes **有**
☐ No**没有**<br>☐
6. 你有多少个孩子？**How many children do you have?**
☐ 1
☐ 2
☐ 3
☐ 4
☐ 5 or more

**7. Age of Children (years)**
☐ 1st Child 第一个孩子的年龄（岁）:
☐ 2nd Child: 第二个的孩子年龄（岁）:
☐ 3rd Child: 第三个的孩子年龄（岁）:
☐ 4th Child 第四个的孩子年龄（岁）:

**8. 族裔 Ancestry**	
☐ 華族 Chinese	
☐ 其他，請表明 Other (please list):	

9. 出生地 Country of birth	
☐ 中國大陸 Mainland China	
☐ 香港 / 澳門 Hong Kong/Macau	
☐ 澳洲 Australia	
☐ 其他，請表明 Other (please specify):	

10. 你在澳洲住了多久？How long have you been living in Australia?	
________年Years ___ 月 Months	

11. 來澳洲之前，你在中國大陸住了多久？How long did you live in Mainland China before moving to Australia?	
________年Years ___ 月 Months	

12. 學歷 Educational Attainment	
☐ 沒有讀過書 No school certificate or other qualification	
☐ 小學畢業 School or intermediate certificate	
☐ 中學畢業 Higher school or leaving certificate	
☐ 文憑畢業 Certificate or diploma	
☐ 大學及以上 University degree or higher	

13. 家裏說什麼語言（可選多項）Language(s) spoken at home (choose all that apply)	
☐ 英文 English	
☐ 普通話 Mandarin	
廣東話 Cantonese	
☐ 其他方言，請表明 Other Chinese dialect (please specify):	
☐ 其他語言 Other:	

14. 每年收入（澳幣）Annual Household Income level (Australian dollars)	

□ 少於 less than $5,000	
□ $5,000-$9,999	
□ $10,000-$19,999	
□ $20,000-$29,999	
□ $30,000-$39,999	
□ $40,000-$49,999	
□ $50,000-$69,999	
□ 多於 $70,000 or more	
□ 我選擇不回答 Rather not answer this question	

**15. 目前工作狀況 Current Working status**	
□ 已退休 Fully retired	
□ 半退休 Partly retired	
□ 全職工作 Full-time	
□ 兼職工作 Part-time	
□ 自僱 Self employed	
□ 在家照顧家人 Home / Family Care	
□ 義工 Unpaid work	
□ 因病在家 Disabled/sick	
□ 無業 Unemployed	
□ 全職學生 Studying	

醫療與健康資料MEDICAL/HEALTH RELATED

**16.** 高度 (厘米) **Height (cm)**	

**17.** 重量 (公斤) **Weight (kg)**	

**18.** 腰围 (厘米) **Waist Circumference (cm)**	

**19. 吸烟習慣 Smoking status**	
□ 有吸烟 Current smoker	
□ 已戒烟 Ex-smoker	
□ 從不吸烟 Never smoker	

**20. 你有被確診以下的疾病嗎？（可選多項）Have you been diagnosed with any of the following conditions? (choose all that apply)**	
□ 心臟病 Heart disease	
□ 高血壓 High blood pressure	
□ 中風 Stroke	

糖尿病 Diabetes	
沒有以上疾病 None of the above	

**21. 你有吃任何控制血脂或血糖的西藥或中藥嗎？Are you taking any medications, including traditional Chinese medicine, to manage blood lipids or blood sugar (prescribed or not prescribed)?**	
有 Yes	
沒有 No	
如果有，請列出你吃的藥If yes please list medications taken:	

**22. 你通常一晚睡幾個小時 Usual sleep hours per night**	
少於 5 or fewer	
6	
7	
8	
9	
10+	
